# Vision impairment in boys recruited to the iREAD study

**DOI:** 10.1186/s13584-025-00667-7

**Published:** 2025-01-27

**Authors:** Jonathan Levine, Ravid Doron, Lisa A. Ostrin, Einat Shneor

**Affiliations:** 1 Department of Optometry, Hadassah Academic College, 9101001 Jerusalem, Israel; 2 College of Optometry, University of Houston, Houston, TX 77004 USA

**Keywords:** Vision impairment, Reduced vision, Refractive error, Public health, Myopia, Hyperopia, Astigmatism, Amblyopia

## Abstract

**Background:**

Uncorrected refractive error is reported to be the most common cause globally of vision impairment in school age children. However, little is known about the extent of uncorrected refractive error in Israel. The purpose of this study was to investigate the prevalence of vision impairment in schoolchildren recruited for the Israel Refraction, Environment, And Devices (iREAD) Study.

**Methods:**

Healthy boys, ages 5–13 years, were recruited to participate in the iREAD Study. Parents first answered a questionnaire to exclude children with a known history of amblyopia, strabismus, or hyperopia. A comprehensive eye exam was then performed. Presenting visual acuity < 6/12 was defined as vision impairment. Myopia and hyperopia were defined as cycloplegic spherical equivalent refraction  ≤ − 0.50 D, and ≥  + 0.50 D, respectively, and astigmatism as ≤ − 0.75 D. Amblyopia was defined as best corrected visual acuity ≤ 6/12 in at least one eye in the absence of any ocular pathology. Descriptive statistics were used to calculate the prevalence of each refractive error and amblyopia.

**Results:**

Two hundred five boys (average age 8.8 ± 1.7 years) presented for a comprehensive eye exam. The prevalence of vision impairment at initial presentation was 22.9% (N = 47), with 16.1% (N = 33) and 6.8% (N = 14) for both eyes and one eye, respectively. Of the children with vision impairment, 36.2% (N = 17) were wearing habitual correction. Of the children with vision impairment, 97.9% (N = 46) had refractive error, with 85.1% (N = 40) being myopic and 12.8% (N = 6) being hyperopic. In addition, 36.2% (N = 17) with vision impairment had astigmatism. Most children with vision impairment (N = 43) achieved good vision with refractive correction. However, amblyopia was observed in 2.0% (N = 4) of the children.

**Conclusions:**

A high prevalence of vision impairment was observed, primarily due to uncorrected or undercorrected refractive error. Children with amblyopia and/or hyperopia presented despite a parent questionnaire to exclude children with these conditions. Findings suggest that many parents are unaware of their children’s visual and refractive status, even for children who already have glasses. In conclusion, improvements to the current system in Israel of vision screenings in first grade should be made to help insure children in need receive adequate follow-up throughout their education.

## Background

Vision impairment is defined by the World Health Organization as “an eye condition that affects the visual system and its vision functions” [1] and includes conditions such as uncorrected refractive errors, cataract, diabetic retinopathy, glaucoma, and age-related macular degeneration. Vision impairment poses a significant global socioeconomic burden, with associated annual global costs of productivity losses estimated to be US$ 411 billion [2]. The leading causes of vision impairment in adults are uncorrected refractive errors and cataracts [3, 4], in both high [5]—and low-income countries [4]. Uncorrected refractive error is the leading cause of vision impairment in children [6]. Varma, et al. estimated that 174,000 preschool children in the USA were visually impaired in 2015, with 69% being due to uncorrected refractive error [6]. These numbers are projected to increase by 26% by the year 2060 [6].Given the significant impact vision has on childhood development, education, employability, and lifelong independence [7–10], it is vitally important to identify and treat children with undiagnosed vision impairment.

Israel has a robust health care system with all citizens being required by law to have health insurance. Mother Child Health Centers provide free pediatric preventive health services from birth to six years of age, as mandated by law [11, 12]. Ophthalmology services are free as part of insurance, and vision screenings are provided by Maternal Child Health Clinics at age four and at schools in the first grade. Despite this, epidemiological studies in Israel have found that many children present to school with a vision impairment [13, 14].

The visual status of school age children in Israel has not been studied in a systematic manner in the past decade, nor has it been studied in the Jerusalem area. The current study reports the prevalence of vision impairment in children in the Jerusalem area who were recruited to the Israel Refraction, Environment, And Devices (iREAD) Study [15]. Findings highlight the need for more comprehensive ocular examinations and follow-up in children.

## Methods

### Participants

Boys from the Jerusalem area, ages 5–13 years, were recruited for a baseline visit for the iREAD study from March 2021 to July 2022 [15–18]. The iRead Study is an ongoing 24-month longitudinal study to assess risk factors for myopia in boys enrolled in three school systems: (1) ultra-Orthodox, (2) religious, and (3) secular. Informed consent was obtained from all children and their parent or legal guardian. The study was approved by the ethics committee of Hadassah College and followed the tenets of the Declaration of Helsinki.

The recruitment strategy involved sending out a link via social media explaining the study and asking several screening questions. Screening questions were aimed at excluding children with a history of ocular or systematic pathology, such as amblyopia. Parents of potential participants first answered the recruitment questionnaire, which asked the following questions:

- Is your child generally healthy?
- Does your child have a lazy eye?
- Does your child have strabismus (squint)?
- Has your child had an eye operation or myopia control treatment?
- Does your child wear spectacles or contact lenses?
  - If yes, does the child wear them for:
    - Distance
    - Near
    - All the time

Children with amblyopia, strabismus, or those wearing glasses for near work (presumed hyperopia) were excluded from the iREAD study to focus on refractive errors that could contribute to the development of myopia. Therefore, parents who answered “yes” to their child having lazy eye or strabismus received a message that their child did not qualify to participate in the study, and their answers were not recorded. The questions regarding spectacle or contact lens wear were included to determine the child’s refractive error and were adopted from a validated questionnaire [19]. Parents who reported that their child wore spectacles all the time or for distance were included based on the assumption that the child was myopic. Parents who reported that their child wore spectacles or contact lenses for near work received a message excluding their child based on the assumption that the child was hyperopic. Children with amblyopia, strabismus, or those wearing glasses for near work (presumed hyperopia) were excluded from the study to focus on refractive errors that could contribute to the development of myopia.

If the parents completed the recruitment questionnaire and were not excluded at that point, they received a phone call inviting them to bring their son to participate in the study.

### Ocular examination

Participation in the study included a full cycloplegic ocular examination. First, monocular and binocular habitual distance visual acuity was tested, with spectacles if the child presented with them. If a child had spectacles but did not bring them to the exam, uncorrected visual acuity was used for presenting visual acuity. A subjective refractive exam was performed, and uncorrected and best corrected visual acuities (VA) were measured. Eyes were then dilated by an ophthalmologist with 1% cyclopentolate (Concept for Pharmacy ltd) and 0.5% tropicamide (Fischer Pharmaceutical Labs). Following dilation, fundus photos were captured and reviewed on the spot by the ophthalmologist. Axial length was measured three times in both eyes (LenStar, Haag-Streit AG, Switzerland), and the average for each eye was calculated. Cycloplegic refractive error was measured in both eyes by autorefraction (VX130, Luneau, France). Three measurements were recorded, and the spherical equivalent refraction (SER) was calculated for each eye. The final spectacle prescription was determined, and children who needed glasses were given a prescription and free glasses.

Vision impairment was defined using the International Classification of Diseases 11 (2018) [20], which classifies distance vision impairment as follows:

- No vision impairment—equal or better than 6/12 (≥ 0.5)
- Mild—visual acuity worse than 6/12 to 6/18 (< 0.5 to ≥ 0.33)
- Moderate—visual acuity worse than 6/18 to 6/60 (< 0.33 to ≥ 0.10)
- Severe—visual acuity worse than 6/60 to 3/60 (< 0.10 to ≥ 0.05)
- Blindness—visual acuity worse than 3/60 (< 0.05)

Each eye was classified as myopic (≤ − 0.50 D) [21], hyperopic (≥ + 0.50 D, significantly hyperopic (≥ + 2.50 D), or emmetropic (+ 0.50 to <  − 0.50 D) based on average cycloplegic spherical equivalent refraction. If a child had emmetropia in one eye and the other myopia or hyperopia, he was classified according to the worse eye. If a child had myopia in one eye and hyperopia in the other, he was classified as myopia + hyperopia. Astigmatism was defined as cylinder > 0.75 D, and significant astigmatism was defined as > 2.50 D.

### Statistical analysis

Assuming a prevalence of binocular visual acuity < 6/12 (0.5) of 16% for Jewish children [14], a precision of 7.5% and a confidence level of 95%, a sample size of 92 was required to determine prevalence of vision impairment. Descriptive statistics were used to calculate the prevalence of amblyopia and each refractive error. Means ± standard deviations are presented.

## Results

For the 205 boys that presented for a comprehensive exam, mean age was 8.8 ± 1.7 years (range: 5.1–13.7 years). Mean uncorrected VA of the total cohort was 0.68 ± 0.36 Snellen decimal. Thirty-nine boys presented with habitual correction, and 4 boys had habitual correction but left their glasses at home. The mean presenting VA (i.e. with habitual correction for those who brought it with them and uncorrected for those without) was 0.75 ± 0.30 decimal.

After refraction, the mean best corrected VA was 0.94 ± 0.14 (range 0.10–1.20 D) decimal. Mean cycloplegic refraction and axial length were − 0.08 ± 1.82 (range − 8.08 to + 8.00 D) D and 23.34 ± 1.14 mm (20.49–28.33 mm) and − 0.11 ± 1.75 D (range − 7.58 to + 8.25 D) and 23.31 ± 1.05 mm (20.59–26.62 mm) for the right and left eyes, respectively. When divided into refractive error categories (based on at least one eye), 35.6% were myopic and 44.9% were hyperopic (Table 1). Of the hyperopic children, 4.9% had significant hyperopia. Thirty percent of the children had astigmatism > 0.75 D, including 2.0% with significant astigmatism > 3.000 D.

**Table 1:** Number of children (N = 205) with emmetropia, myopia, or hyperopia and astigmatism in either one or both eyes based on cycloplegic spherical equivalent refraction (SER)

	Monocular N	Binocular N	Total N (%) [CI]
Emmetropia (SER − 0.50 to + 0.50 D)	0	38	38 (18.5%) [13.8–24.4%]
Myopia (SER ≤ − 0.50 D)	18	55	73 (35.6%) [29.4–42.4%]
Total Hyperopia (SER ≥ + 0.50 D)	27	65	92 (44.9%) [38.2–51.7%]
Hyperopia (SER ≥ + 0.50 D to 2.50 D)	21	61	82 (40%) [33.5–46.8%]
Significant Hyperopia (SER ≥ 2.50 D)	6	4	10 (4.9%) [2.7–8.8%]
Myopia + Hyperopia	n/a	2	2 (1%) [0.3–3.5%]
Total Astigmatism	28	33	61 (30.0%) [28.5–31.5%]
Astigmatism (cyl ≥ 0.75 D)	28	29	57 (27.8%) [23.9–36.3%]
Significant astigmatism (cyl ≥ 3.00 D)	2	2	4 (2.0%) [0.7–4.9%]

*CI* 95% Confidence Interval

**Fig. 1:**
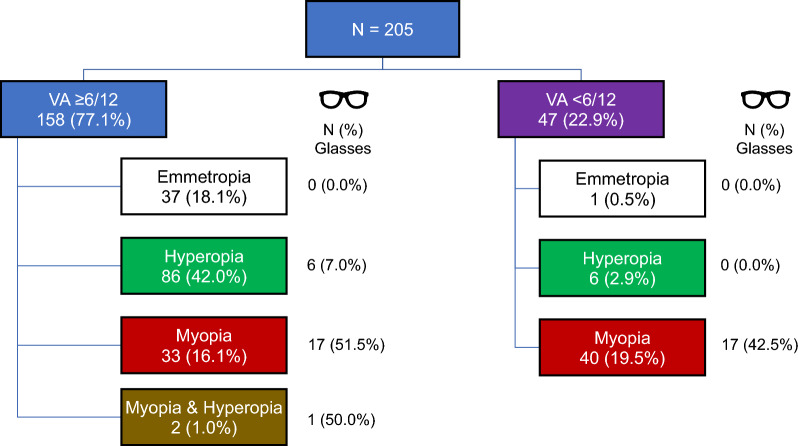
Distribution of children with normal presenting vision (VA ≥ 6/12) and vision impairment (VA < 6/12) according to refractive error category. The numbers and percentages in the rectangles refer to the number of children in each refractive category, and the numbers and percentages under the glasses icon refer to the number of children who presented with glasses

The prevalence of vision impairment at presentation was 22.9% (N = 47) with 16.1% (N = 33) and 6.8% (N = 14) for both eyes and one eye, respectively (Fig. 1, Table 2). Among these children, 14.9% (N = 7) had mild impairment, 59.6% (N = 34) had moderate impairment, 8.8% (N = 5) had severe impairment, and 1.8% (N = 1) were classified as blind. Notably, 36.2% (N = 17) of children with vision impairment presented with glasses but still had reduced vision.

**Table 2:** Prevalence of children presenting with vision impairment in one or both eyes (VA in decimals). [CI: 95% Confidence Interval]

	Monocular N	Binocular N	Total N (%) [CI]
Any vision impairment	14	33	47 (22.9%) [17.7–29.2%]
Mild vision impairment (VA < 0.5 to ≥ 0.33)	5	2	7 (3.4%) [1.7–6.9%]
Moderate vision impairment (VA < 0.33 to ≥ 0.10)	8	26	34 (16.6%) [12.1–22.3%]
Severe vision impairment (VA < 0.10 to ≥ 0.05)	1	4	5 (2.4%) [1.1–5.6%]
Blindness vision impairment (VA < 0.05)	0	1	1 (0.5%) [0.09–2.7%]

Of the 47 children presenting with vision impairment, 97.9% of the children (N = 46) also had refractive error; 85.1% (N = 40) were myopic, 12.8% (N = 6) were hyperopic, and 2% (N = 1) emmetropic (Fig. 1, Table 3). In addition, 36.2% (N = 17) of the children with vision impairment had astigmatism. Of the myopic children, only 42.5% (N = 17) presented with glasses, while none of the hyperopic or amblyopic children presented with glasses. Aside from four children with amblyopia, all achieved visual acuity > 6/12 with subjective refraction.

**Table 3:** Prevalence of refractive errors of children with vision impairment

	Monocular N	Binocular N	Total N (%) [CI]
Emmetropia (SER − 0.50 to + 0.50 D)	0	1	1 (2.1%) [0.4–11.1%]
Myopia (SER ≤ − 0.50 D)	5	35	40 (85.1%) [72.3–92.6%]
Total Hyperopia (SER ≥ + 0.50 D)	3	3	6 (12.8%) [6.0–25.2%]
Hyperopia (SER ≥ + 0.50 D to 2.50 D)	1	1	2 (4.3%) [1.2–14.3%]
Significant Hyperopia (SER ≥ 2.50 D)	1	2	4 (8.5%) [3.4–19.9%]
Total Astigmatism	5	12	17 (36.2%) [24.0–50.5%]
Significant astigmatism (≤ − 3.00D)	0	1	1 (2.1%) [0.4–11.1%]
Astigmatism (≤ − 0.75D)	5	10	15 (31.9%) [20.4–46.2%]
Only Astigmatism (Emmetropia)	1	0	1 (2.1%) [0.4–11.1%]

*CI* 95% Confidence Interval

Four children (2%) presented with amblyopia, despite their parents having answered a questionnaire that would have excluded them if they had known. Of these children, three had bilateral amblyopia and one had monocular amblyopia. The amblyopia was likely caused by high hyperopia (Table 4, child 1), high astigmatism (child 2) or anisometropia (child 3). One child (4) had visual acuity that did not correlate with his low refractive error suggesting the presence of other pathology. None of these children had been prescribed glasses in the past. All four children were referred to pediatric ophthalmology for further evaluation and treatment.

**Table 4:** Clinical findings of children with amblyopia

	Age (years)	PVA OD (dec)	Cyclo-Sphere refraction OD (D)	Cyclo-Cylinder refraction OD (D)	BCVA OD (dec)	PVA OS (dec)	Cyclo-Sphere refraction OS (D)	Cyclo-Cylinder refraction OS (D)	BCVA OS (dec)
1	5.1	0.01	+ 7.50	− 3.25	0.1	0.01	+ 7.50	− 3.25	0.1
2	5.3	0.3	− 0.08	− 2.67	0.3	0.40	+ 0.17	− 2.25	0.4
3	8.2	0.4	+ 2.25	− 0.75	0.5	0.50	+ 0.75	− 0.92	0.8
4	9.1	0.1	+ 0.33	− 0.42	0.25	0.10	+ 0.17	− 0.50	0.25

*PVA* Presenting visual acuity; *BCVA* Best corrected visual acuity; *OD* Right eye; *OS* left eye; *D* diopter; *dec* Snellen decimal

To compare with previous studies in Israel, vision impairment was also defined as ≤ 0.5 (instead of < 0.5) [22]. Using this definition, 27.8% of the children presented with mild to moderate vision impairment in at least one eye. Of those children, 29.8% (N = 17), 59.6% (N = 34), 8.8% (N = 5), 1.8% (N = 1), had mild, moderate, severe, and blindness vision impairment, respectively.

## Discussion

The examination of 205 boys revealed a significant prevalence of vision impairment, primarily due to uncorrected refractive errors, including myopia, hyperopia, and astigmatism. Our findings showed that almost all the children who had vision impairment were ameliorated with accurate refractive correction. Early detection and intervention could restore near-optimal vision for a majority of affected individuals. Additionally, the identification of amblyopia in several participants, a condition previously unnoticed by parents, underscores the essential role of regular professional eye examinations in detecting and managing eye health issues early on. Overall, these results highlight gaps in provision of care and the imperative need for enhanced accessibility and parent education regarding eye care services for the pediatric patient population in the Jerusalem area.

For the total population of boys examined in this study, the prevalence of myopia was 35.6%. This high prevalence of myopia falls within trends observed in global studies and aligns with findings from previous research in similar ages, conducted in Israel [23], For example, the prevalence of myopia is 34.7% in children ages 5–14 years in the USA, Southern California [24]; 38.1–57.1% in China [25, 26]; and 49.7% in Sweden [27]. In contrast, a study from New Delhi, India found a myopia prevalence of 7.4% [28], and a rural population in Iran demonstrated even lower rates of myopia (2.60%) [29]. These significant differences may stem from genetic, environmental, and lifestyle variations such as outdoor activity and educational demands [15, 16, 23, 30–33], which serve as significant risk factors for the development of myopia in children. In addition, children who live in urban areas have been shown to have a higher rate of myopia [34–36]; children in the current study lived in Jerusalem, a large urban city.

Vision impairment in children represents a significant public health concern, marked by varying prevalence rates across different countries and age groups**.** In the current study, the observed 22.9% prevalence of vision impairment for a sample of children in Israel, predominantly due to refractive errors like myopia, significantly surpasses global averages (4.34–10.19% [37]) and aligns with prior studies within Israel [13, 22, 38, 39].

The prevalence of vision impairment reported in this study notably exceeded those from neighboring countries like Saudi Arabia (11.86% [40]) and Sudan. (4.40% [41]). In contrast, the prevalence of vision impairment in this study was more comparable to China (19.3%), which has also high rates of myopia [42, 43]. Additionally, the fact that the prevalence of vision impairment in Israel surpasses these populations with high rates of myopia suggests that factors beyond myopia itself contribute to elevated rates of vision impairment in Israel [44–50]. This disparity underscores the urgent need for comprehensive public health strategies to address this issue within Israel [13, 22, 38, 39, 51, 52].

In contrast to the global average, the prevalence of vision impairment in this study is similar to previous studies in Israel. A study of 1975 first and eighth grade children in Northern Israel, found that 23.3% had presenting visual acuity worse than 6/12 in both eyes, including those who presented with spectacles [22]. Similarly, a survey of 917 third grade children in central Israel, which used a different definition of vision impairment (presenting binocular visual acuity worse than 6/6) found that 41% of the children had vision impairment, including children who presented with spectacles [13]. Additionally, a recent study examining preschool vision screening for children ages 3–6 years in Israel reported a referral rate of 23.0% from screening tests conducted at community-based Mother Child Health Centers, indicating a significant detection rate of potential visual issues in this younger cohort [53].

The high rate of vision impairment in Israel is surprising, considering the robust national health care system in Israel. Visual acuity screening at ages 3 and 5 are part of the national preventive health services offered at Maternal Child Health Clinic, and distance visual acuity screening in first grade is part of the school health services funded by the national government. However, the Maternal Child Health Clinic exams are performed by nurses, rather than eye care professionals, and compliance with Maternal Child Health Clinic visits at this age is very low in many areas of the country [53]. Although vision screenings are performed at Maternal Child Health Clinics, the data presented in this study suggest that some vision impairments, including amblyopia and refractive errors, may not be detected through these screenings. Alternatively, the children may not have participated in the vision screening program. Only about 25% of children present for the vision screening exam [54] and only about half of these receive a comprehensive eye exam [53]. A more detailed assessment of the sensitivity and specificity of these screenings is needed to better understand their effectiveness. Furthermore, myopia often manifests at a later age than those screened by the Maternal Child Health Clinic and subsequent to the first grade in-school exam. It is also possible that when children fail vision screenings, parents do not comply with the referral for a complete eye exam. While the current study focuses on older children, it is important to note that Israel has recently introduced an amblyopia screening program in nurseries for 3-year-olds. Although this initiative is still being rolled out, it represents a significant step forward in early detection of vision problems. This is supported by a study that showed that only 50% of children who failed the vision screening at the Maternal Child Health Clinic received a full eye exam [53]. In addition, even those who do comply with an initial eye exam may not adequately follow up for subsequent vision screenings. Indeed, 43% of the myopic children in this study who presented with vision impairment already had glasses prescribed by an eye care provider. Given these gaps, it is not surprising that our study identified 40 children with myopia and impaired vision despite the presence of a nationally mandated vision screening system. Systemic barriers, including limited access to comprehensive pediatric eye care and the lack of a cohesive data management system that tracks screening, referral, and treatment, may contribute to the high prevalence of untreated vision impairment observed in this study. Strengthening these systems could improve the detection and treatment of vision impairment in children.

Moreover, the study highlights the critical role of professional eye examinations in the early identification and management of ocular pathologies, including amblyopia, which was previously undetected in 2% of participants. This finding underscores the inadequacies of relying solely on parental assessments to detect vision impairment and the importance of routine comprehensive ocular evaluations by skilled practitioners.

Another potential obstacle to comprehensive vision exams for children is the limited access to pediatric eye care in Israel. With only approximately 55 pediatric ophthalmologists in the country and approximately 1.3 million children under the age of eight [54, 55], there exists a significant disparity between the demand for services and the available resources. Optometrists and general ophthalmologists could play a crucial role in conducting pediatric vision exams; however, scope-of-practice restrictions on optometrists and the reluctance of ophthalmologists to work with the pediatric population pose challenges. In Israel, optometrists are prohibited from using diagnostic pharmaceuticals, limiting their ability to examine children effectively. Moreover, many Israeli ophthalmologists feel insufficiently equipped or experienced to perform the necessary tests for the pediatric population, particularly during the critical period before 8 years of age [56].

The socioeconomic implications of vision impairment are profound, with potential to exacerbate economic burdens and contribute to poverty [57]. Moreover, significant uncorrected refractive errors have been linked to diminished visuo-cognitive abilities, reading skills, and visual attention in young children [8, 9]. This relationship emphasizes the need for public health strategies and policies that support accessible and affordable eye care. Notably, preschool children with uncorrected hyperopia (> 4.00 D) or astigmatism (> 2.00 D) show inferior cognitive test performance compared to their emmetropic peers, although when these children were given glasses and retested after six weeks, their performance significantly improved, showing the positive impact of correcting vision problems on cognitive abilities [8, 10]. This suggests that timely intervention can mitigate the adverse effects of refractive errors on cognitive functions and educational performance.

In addition to the socioeconomic implications of vision impairment in general, the financial impact of amblyopia specifically is significant, with income losses related to the condition surpassing $7 billion annually in the United States alone, far exceeding estimated treatment costs of $341 million [58–60]. In the current study, four participants (2%) presented with undiagnosed amblyopia. This rate is similar to that found in previous studies in other countries of children who had not been screened [60–63]. In Israel, the prevalence of amblyopia in Israel varies depending on the population studied. In 17-year-old military pre-recruits, amblyopia prevalence was shown to range from 0.8 to 1.5% [64, 65]. However, studies in pre-recruits may not reflect the accurate prevalence of amblyopia in Israel, since the cohort does not include most Israeli Arabs or teenagers with disabilities, both of whom are exempt from army service, nor those successfully treated for amblyogenic conditions in childhood. One study addressed this issue in 8-year-old children and found the prevalence of amblyopia to be 1% in children who had been screened in infancy and 2.6% in children not screened in infancy [66].

Given the high prevalence of myopia detected within the population, annual vision exams for children are imperative. We propose two primary approaches to facilitate these exams: (1) Implementing annual vision screening tests in schools for every grade or (2) offering comprehensive exams as part of the national health system. Both have advantages and disadvantages. Annual vision screening offers extensive access to a large number of children, ensuring comprehensive coverage. Currently, school nurses conduct these screenings using visual acuity charts, but there is a need for personnel with more training in ocular conditions and the use of screening methods that have higher specificity and sensitivity. Furthermore, screening is only offered in first and eighth grade, potentially missing children who develop refractive errors in grades 2–7. Another barrier to vision screening is that parents of the children who fail may not adhere with the recommendation for a comprehensive exam [53].

The other possibility is to incorporate a full vision examination once a year, as part of the national health insurance coverage. This approach guarantees higher sensitivity and specificity. The lack of Health care practitioners is the major barrier to this method. At present, in Israel only ophthalmologists are permitted to use cycloplegic diagnostics, which is essential for examining young children. Despite this, most ophthalmologists report that they are not comfortable performing refraction and prescribing glasses to children [56]. Expanding the scope of practice for optometrists to include cycloplegia, as practiced in many Organization for Economic Co-operation and Development (OECD) OECD countries, is another potential solution. Indeed, greater optometric scope of practice is inversely correlated with the prevalence of vision impairment [67]. The current curriculum in Israel already prepares optometry students in the use of diagnostic pharmaceuticals and the examination of children, making this a feasible goal [68].

Another health policy issue is parental health literacy. Despite the broad coverage and potential effectiveness of school-based screenings, there is a significant gap in adherence to referrals among parents, pointing out the need for improved parental education and engagement strategies [53, 69]. By enhancing understanding and cooperation from parents, we can significantly increase the effectiveness of both school-based and national health coverage approaches of vision screenings, ensuring that children receive the necessary follow-up care [70–72].

The current study is subject to the following limitations. Primarily, the research was confined to a narrow demographic—boys aged 5 to 14 in Jerusalem area of Israel—potentially limiting the applicability of our findings across the entire pediatric population, including girls and children from diverse socio-economic and ethnic backgrounds. Moreover, the reliance on parental reports for historical vision-related health data could have introduced recall bias. A limitation of the current study is the lack of data on whether participants had undergone previous vision screenings at age 4 or in first grade through national screening programs. Future studies should aim to collect this information to better understand the gaps in screening and follow-up care.

Despite the study's reliance on a questionnaire-based screening process intended to filter out children with amblyopia and hyperopia, the voluntary participation aspect could have biased our sample towards individuals with undiagnosed conditions or concerns about their vision. Nonetheless, the consistency of our findings with prior research [60–63] suggests that the observed prevalence rates of uncorrected vision issues and amblyopia may not deviate significantly from those in the broader population. It is possible that our exclusion criteria were not entirely effective in filtering out all hyperopic children, as indicated by the inclusion of participants who were wearing glasses and had been diagnosed with hyperopia. Another potential bias arises from the study’s protocol requiring participants to commit to a two-week period of wearing a monitoring watch device [15, 16], possibly excluding children unwilling or unable to comply. Ideally, a cross-sectional methodology conducted in a neutral setting, such as schools, would minimize selection bias and better reflect the prevalence of vision issues in the general population. In addition, the data were collected from three different types of schools (ultra-Orthodox, religious, and secular); however, a detailed breakdown by school type was not included in this analysis, as the study focused on understanding the overall prevalence of vision impairment in this population. Future publications may explore these differences in more detail.

## Conclusions

This study emphasizes the urgency for enhanced public health initiatives aimed at promoting eye care awareness amongst parents and improving vision screening in schools, given the high prevalence of vision impairments in children. Highlighting the critical importance of early detection, accurate diagnosis, and prompt intervention, the data underscores the necessity for improved access to comprehensive eye care services, including effective measures for identifying and addressing conditions such as refractive errors and amblyopia. Furthermore, the study sheds light on the challenges associated with ensuring adherence to and access to expert care, emphasizing the need for systemic reforms within the Israeli healthcare system to enhance screening and treatment for children with vision impairments. Moving forward, future research should focus on evaluating vision impairments and developing intervention strategies through longitudinal studies across diverse demographic groups. By addressing these issues collaboratively, we can utilize insights gained from data across varied genetic, environmental, and lifestyle factors to significantly reduce the prevalence of vision impairments among children globally.

## Data Availability

The datasets during and/or analysed during the current study available from the corresponding author on reasonable request.

## Abbreviations

iREAD Study: For the Israel Refraction, Environment, And Devices Study
SER: Spherical equivalent refraction
VA: Visual acuity
CI: 95% Confidence Interval
PVA: Presenting visual acuity
BCVA: Best corrected visual acuity
Dec: Snellen decimal
